# Reply to Orlhac, F.; Buvat, I. Comment on “Ibrahim et al. The Effects of In-Plane Spatial Resolution on CT-Based Radiomic Features’ Stability with and without ComBat Harmonization. *Cancers* 2021, *13*, 1848”

**DOI:** 10.3390/cancers13123080

**Published:** 2021-06-21

**Authors:** Abdalla Ibrahim, Turkey Refaee, Sergey Primakov, Bruno Barufaldi, Raymond J. Acciavatti, Renée W. Y. Granzier, Roland Hustinx, Felix M. Mottaghy, Henry C. Woodruff, Joachim E. Wildberger, Philippe Lambin, Andrew D. A. Maidment

**Affiliations:** 1 The D-Lab, Department of Precision Medicine, GROW—School for Oncology, Maastricht University, 6200 Maastricht, The Netherlands; t.refaee@maastrichtuniversity.nl (T.R.); s.primakov@maastrichtuniversity.nl (S.P.); h.woodruff@maastrichtuniversity.nl (H.C.W.); philippe.lambin@maastrichtuniversity.nl (P.L.); 2 Department of Radiology and Nuclear Medicine, Maastricht University Medical Centre+, 6200 Maastricht, The Netherlands; felix.mottaghy@mumc.nl (F.M.M.); j.wildberger@mumc.nl (J.E.W.); 3 Division of Nuclear Medicine and Oncological Imaging, Department of Medical Physics, University Hospital of Liège and GIGA CRC-In Vivo Imaging, University of Liège, 4000 Liege, Belgium; rhustinx@ulg.ac.be; 4 Department of Nuclear Medicine and Comprehensive Diagnostic Center Aachen (CDCA), University Hospital RWTH Aachen University, 52074 Aachen, Germany; 5 Department of Diagnostic Radiology, Faculty of Applied Medical Sciences, Jazan University, Jazan 45142, Saudi Arabia; 6 Department of Radiology, Perelman School of Medicine, University of Pennsylvania, Philadelphia, PA 19104, USA; Bruno.Barufaldi@pennmedicine.upenn.edu (B.B.); racci@pennmedicine.upenn.edu (R.J.A.); Andrew.Maidment@pennmedicine.upenn.edu (A.D.A.M.); 7 Department of Surgery, GROW—School for Oncology, Maastricht University Medical Centre+, 6200 Maastricht, The Netherlands; r.granzier@maastrichtuniversity.nl

We would like to thank Orlhac and Buvat [1] for their commentary on our article [2]. Orlhac and Buvat present the opinion that we “misused” ComBat harmonisation to assess radiomic features in a computed tomography (CT) phantom by evaluating the phantom as a whole. They state that we must apply ComBat harmonisation separately to each layer of the phantom, akin to restricting a radiomics study to either liver or tumour. However, the main aim of our work [2] was not to address a specific radiomics task, but to use CT phantom data to evaluate the robustness of 91 radiomics features to changes in voxel size, either alone or with two harmonisation methods—interpolation and ComBat.

The application of the ComBat method of Johnson [3] to radiomics, proposed by Fortin et al. [4], arose after its initial application to genomics. Johnson sought to harmonise data that were divided into “batches”, “samples”, and “genes”. ComBat “incorporates systematic batch biases common across genes in making adjustments, assuming that phenomena resulting in batch effects often affect many genes in similar ways (i.e., increased expression, higher variability, etc.)” [3]. In the application of ComBat to radiomics, we and Orlhac [5] are in agreement that the radiomic features are Johnson’s genes, and that the scans are Johnson’s batches. Thus, the difference comes down to the definition of the sample. Johnson proposed the definition of a sample as being, for example, a patient. By contrast, Orlhac and Buvat state “that all measurements grouped in the same batch are *equally* affected by the imaging protocol” [1] (emphasis added), and thus propose that the sample must be a specific texture, for example, “liver or tumour”, based on the assumption that various textures are affected differently. We believe that this is overly prescriptive. In our usage, the sample is the phantom, which is intended to represent a range of tissue types, because we sought to understand how acquisition differences affect each measure over a range of materials [2]. This is consistent with the use of Combat by Fortin et al. [4].

Consider a simple example—namely that of the first order mean. The phantom in question has 10 layers representing different tissues, including several layers that have a uniform single material. In Figure 1, we show a plot of the paired values of the mean for a single layer and for the whole phantom. By default, all CT scanners use, at a minimum, a two-point calibration of the Hounsfield units (HU), typically performed daily. Nevertheless, CT scans are subject to both stochastic noise arising from the X-radiation and electronic noise in the CT scan, and non-stochastic sources of error in the CT systems, such as reconstruction artefacts. However, due to the calibration, the average HU values of a given material in the phantom will be nearly identical in any two scans, regardless of pixel size, especially when averaged over large regions. Figure 1a shows the results for a single layer: they are not strongly correlated, nor should they be correlated if the layer represents a single material or a simple admixture of materials. By contrast, in Figure 1b, we show the results for analysing all phantom layers. As expected, the results are highly correlated since the phantom spans a range of materials. In Figure 2a,b, we show the Concordance Correlation Coefficient (CCC) [6] value for the grayscale mean pairwise across the seven scans, CCR-2-001 to CCR-2-007, considered in our paper. Note that, in analysing a single layer (Figure 2a), we see a moderate to no correlation. This arises directly from the physics of imaging objects with limited material differences; the average HU should only vary by stochastic noise and non-stochastic errors. When we analyse all layers (Figure 2b), all scans show high correlation with each other, as expected. Of note, Orhlac and Buvat used ROIs that were smaller in volume than ours, which only serves to increase the stochastic noise, and leads to even more false correlations.

That said, the message of our paper was that ComBat harmonisation is not a fix-all. Rather, we argued that one should first apply harmonisation steps that directly address physical differences in the acquisition of the images. Fundamental imaging physics dictates that differences such as voxel size, slice thickness, mAs, dose, and kV can profoundly impact the appearance of images. At least some of these factors, for example voxel size or slice thickness, can be readily harmonised through appropriate and direct image processing, such as resampling. In our paper, we demonstrate that *sinc* interpolation is superior to pixel replication (nearest neighbour) and other simple interpolation schemes, and that downsampling (harmonisation to a coarser resolution) to a common spatial resolution is superior to upsampling. These have simple and obvious physical explanations. However, interpolation to a common pixel size is also not a fix-all. Most importantly, we showed that, regardless of the method applied, a reproducibility analysis is required to select reproducible and harmonisable features.

We have also repeated our analysis layer by layer, as recommended by Orlhac and Buvat [1], using both parametric and non-parametric ComBat forms, and the results do not change the conclusions of our paper. As suggested, we re-analysed the same scans (CCR-2-001 and CCR-2-007) using 16 cubic volumes of interest (2 × 2 × 2 cm^3^) per layer. In Table 1, we assess the reproducibility of radiomic features before and after ComBat harmonisation for each layer separately using the cut-off (CCC > 0.9). Indeed, the number of reproducible features before and after ComBat harmonisation differ when analysed per layer (Table 1). These results reinforce our original message, that assessing the reproducibility of features with various harmonisation methods for each radiomic task is essential. Orlhac and Buvat took the additional step of calculating the CCC for all the layers after applying ComBat separately for each layer. This presumes a task for which tissue classification or segmentation is applied before ComBat harmonisation. This is task dependent: for example, Verma et al. [7] considered analysis of grey matter and white matter both separately and jointly, but found no difference in performance.

Orlhac and Buvat also state that the definition of the design matrix of covariates affects the outcome of Combat [1]. We agree. The aim of the design matrix of the biologic covariates in ComBat is to preserve biologic information while harmonising the features [3,8,9]. However, as we have stated [2], we performed this study to examine the impact of pixel interpolation on radiomic features in a phantom, and no biologic covariates were appropriate for our study. We clearly state in the discussion that anthropomorphic phantom scans provide some evidence of the reproducibility of features, but that they cannot completely represent features extracted from human images, and human or cadaveric reproducibility studies are encouraged when ethical.

In summary, we disagree with the statement of Orlhac and Buvat that we “misused” Combat [1]. First, their method of application to a specific material (or in the case of the phantom, a single layer) will not express the full impact of the underlying imaging physics, which we were trying to elicit in our study. Second, by choosing ROI sizes that are sensitive to stochastic noise, Orlhac and Buvat run the risk of overfitting image noise and producing false correlations. Third, Orlhac and Buvat suppose that all radiomic tasks require the same definition of the “sample” be used. For this, we fundamentally disagree; the choice of sample depends upon the task. We do agree with Orlhac and Buvat that the design matrix can affect the outcome of Combat. Finally, it is worth noting that, as described in our paper, we used Pyradiomics version 2.1.2, which has 91 features, and Orlhac used Pyradiomics version 3.0.0, which has 93 features; this accounts for the difference in features between our work and Orlhac [1].

Thus, the message of our study [2] remains unchanged: (1) image interpolation is a useful harmonisation method to address variations in pixel spacing; (2) ComBat harmonisation was of added value in almost all scenarios; (3) the effects of interpolation and ComBat on the reproducibility of radiomic features is dependent on the data being analysed; (4) neither interpolation nor Combat is a fix-all; and (5) regardless of the harmonisation method applied, study data should be analysed to identify *reproducible features*, and used to help interpret and generalise radiomic models developed with these features.

## Figures and Tables

**Figure 1:**
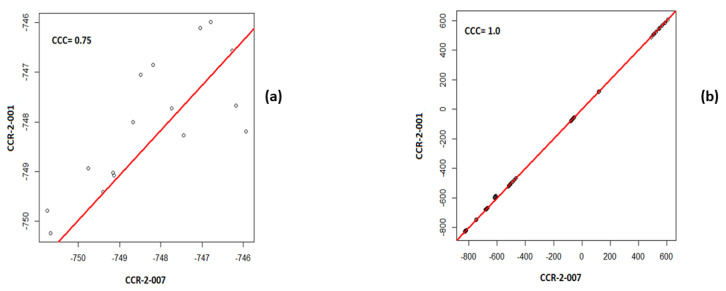
Pairwise plot of the first order mean values with the CCC for (**a**) a single layer of the phantom (ABS-040), (**b**) all layers of the phantom, for the scans CCR-2-001 and CCR-2-007.

**Figure 2:**
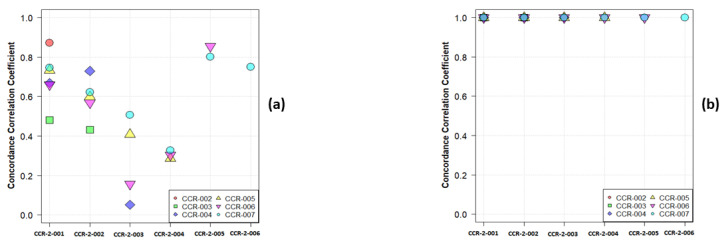
The pairwise CCC values for the first order mean values across 7 scans for (**a**) a single layer of the phantom (ABS-040), (**b**) all the layers.

**Table 1:** The number of reproducible radiomic features for the different phantom layers between scan CCR-2-001 and CCR-2-007.

Phantom Layer	Number (%) before ComBat Harmonisation	Number (%) after ComBat Harmonisation	
		**Parametric**	**Non-Parametric**
ABS-020	0 (0.0%)	3 (3.3%)	3 (3.3%)
ABS-030	0 (0.0%)	1 (1.1%)	0 (0.0%)
ABS-040	0 (0.0%)	3 (3.3%)	3 (3.3%)
ABS-050	3 (3.3%)	14 (15.4%)	9 (9.9%)
Wood	27 (29.7%)	38 (41.2%)	36 (39.6%)
Rubber	2 (2.2%)	36 (39.6%)	31 (36.3%)
Dense Cork	6 (6.6%)	26 (28.6%)	24 (26.4%)
Acrylic	6 (6.6%)	32 (35.2%)	32 (35.2%)
Cork	7 (7.7%)	42 (46.2%)	35 (38.5%)
Resin	22 (24.2%)	44 (48.4%)	41 (45.1%)
